# Prognostic significance of HER2-low status in HR-positive/HER2-negative advanced breast cancer treated with CDK4/6 inhibitors

**DOI:** 10.1038/s41523-023-00534-1

**Published:** 2023-04-17

**Authors:** Emma Zattarin, Daniele Presti, Luigi Mariani, Caterina Sposetti, Rita Leporati, Alice Menichetti, Chiara Corti, Chiara Benvenuti, Giovanni Fucà, Riccardo Lobefaro, Francesca Ligorio, Leonardo Provenzano, Andrea Vingiani, Marta Del Vecchio, Gaia Griguolo, Marianna Sirico, Ottavia Bernocchi, Antonio Marra, Paola Zagami, Elisa Agostinetto, Flavia Jacobs, Pierluigi Di Mauro, Andrea Esposito, Carlo Alberto Giorgi, Luca Lalli, Laura Boldrini, Pier Paolo Berton Giacchetti, Ambra Carnevale Schianca, Valentina Guarneri, Rebecca Pedersini, Agnese Losurdo, Alberto Zambelli, Daniele Generali, Carmen Criscitiello, Giuseppe Curigliano, Giancarlo Pruneri, Filippo de Braud, Maria Vittoria Dieci, Claudio Vernieri

**Affiliations:** 1 Department of Medical Oncology, Fondazione IRCCS Istituto Nazionale dei Tumori, Milan, Italy; 2 Unit of Clinical Epidemiology and Trial Organization, Fondazione IRCCS Istituto Nazionale dei Tumori, Milan, Italy; 3 Oncology 2, Istituto Oncologico Veneto IOV - IRCCS, Padova, Italy; 4 Division of Early Drug Development for Innovative Therapies, IEO, European Institute of Oncology IRCCS, Milan, Italy; 5 IRCCS Humanitas Research Hospital, via Manzoni 56, 20089 Rozzano, Milan Italy; 6 Department of Biomedical Sciences, Humanitas University, Via Rita Levi Montalcini 4, 20072 Pieve Emanuele, Milan Italy; 7 IFOM ETS, the AIRC Institute of Molecular Oncology, Milan, Italy; 8 Pathology Department, Fondazione IRCCS Istituto Nazionale dei Tumori, Milan, Italy; 9 Department of Oncology and Hemato-Oncology, University of Milan, Milan, Italy; 10 Division of Pharmacy, Fondazione Istituto di Ricovero e Cura a Carattere Scientifico (IRCCS) Istituto Nazionale dei Tumori, Milan, Italy; 11 Department of Surgery, Oncology and Gastroenterology-DiSCOG, University of Padova, Padova, Italy; 12 Department of Medical Oncology, IRCCS Istituto Romagnolo per lo Studio dei Tumori (IRST) “Dino Amadori”, 47014 Meldola, Italy; 13 Farmacia Ospedaliera ASST Cremona, Viale Concordia 1, Cremona, Italy; 14 Breast Medicine Service, Department of Medicine, Memorial Sloan Kettering Cancer Center, New York, USA; 15 Institut Jules Bordet and l’Université Libre de Bruxelles, Bruxelles, Belgium; 16 Medical Oncology Unit, ASST Spedali Civili, Brescia, Italy; 17 Breast Cancer Unit & Translational Research Unit, ASST Cremona, Cremona, Italy; 18 Department of Medical, Surgery and Health Sciences, University of Trieste, 34147 Trieste, Italy

**Keywords:** Breast cancer, Tumour biomarkers

## Abstract

Whether Human Epidermal growth factor Receptor 2 (HER2)-low status has prognostic significance in HR + /HER2- advanced Breast Cancer (aBC) patients treated with first-line Endocrine Therapy plus CDK 4/6 inhibitors remains unclear. In 428 patients evaluated, HER2-low status was independently associated with significantly worse PFS and OS when compared with HER2-0 status. Based on our findings, HER2-low status could become a new prognostic biomarker in this clinical setting.

HER2-low breast cancer (BC), as defined by an immunohistochemistry (IHC) score for HER2 of 1 + , or 2+ with negative in situ hybridization (ISH), accounts for ~60% of all BCs classified as HER2-negative, and it includes a substantial proportion of patients historically classified as bearing Hormone Receptor-positive (HR) + /HER2-negative BC or triple-negative BC^1^. HER2-low BC has been recently proposed as a distinct biological and clinical BC entity^1^. However, preclinical and clinical evidence supporting this notion is still poor, also due to conflicting results of clinical studies published so far^2–9^, and which may in part derive from the fact that the prognostic significance of HER2 status has been evaluated in heterogeneous clinical contexts, including patients with different tumor stages, or treated with different types of therapies.

Recently, the antibody-drug conjugate (ADC) Trastuzumab-deruxtecan (T-DXd) significantly improved progression-free survival (PFS) and overall survival (OS) when compared with standard chemotherapy in pre-treated patients with HER2-low advanced BC (aBC), the majority of whom had HR + /HER2-low aBC^10^. While the biological and clinical significance of HER2 status remains unclear, these results have clearly established HER2-low status as a target of a new generation of antibody-drug conjugates (ADCs), such as T-DXd.

The combination of endocrine therapy (ET) and CDK4/6 inhibitors (CDK4/6i) is the standard-of-care, first-line therapy for the vast majority of HR + /HER2-negative aBC patients^11^. However, with the exception of a few small studies^3–9^, the prognostic impact of HER2 status (i.e., low vs. 0) in HR + /HER2-negative aBC patients treated with ET + CDK4/6i remains unclear. Here, we conducted a multicentric study to investigate the association between HER2 status (low vs. 0) and the PFS or OS of HR + /HER2- aBC patients treated with first-line ET + CDK4/6i.

A total of 428 patients treated with first-line ET + CDK4/6i between January 2015 and April 2022 were included in this study; of these, 269 (62.8%) patients had HER2-low disease, while the 159 (37.2%) patients had HER2-0 BC. Patients’ characteristics are displayed in Supplementary Table 1. Palbociclib, Ribociclib and Abemaciclib were prescribed to 291 (68%), 91 (21.3%) and 46 (10.7%) patients, respectively. HER2 status (low vs. 0) did not show a significant association with relevant clinical and tumor-related variables (Supplementary Table 1). After tumor progression to ET + CDK4/6i therapy, patients with HER2-low received a higher number of subsequent lines of systemic therapy (average in HER2-low: 1.47 ± 1.77; average in HER2-0: 1.24 ± 1.49, *p* < 0.0001) (Supplementary Table 1). None of patients with HER2-low disease (0%) received T-DXd as a subsequent line of therapy.

None of patients with HER2-low disease (0%) received T-DXd as a subsequent line of therapy.

With a median follow-up of 36 months, and with the cut-off date on June 17th 2022, median patient PFS in the whole patient cohort was 26 months. Patients with HER2-low disease had significantly worse PFS when compared to patients with HER2-0 BC (median PFS [range]: 23.6 months [18.9–28.1] vs. 32.3 months [26.1-NA]; *p* = 0.014) (Fig. 1). Multivariable analysis adjusting the impact of HER2 status on PFS for clinically relevant covariates (age, ERα expression, Ki-67 expression, number of metastatic sites, disease-free interval [DFI], de novo metastatic disease, ECOG performance status [PS], liver metastases and type of ET) confirmed an independent and statistically significant association between HER2-low status and worse PFS (adjusted hazard ratio [aHR] 1.42; 95% confidence interval [CI]:1.07–1.89; *p* = 0.0163) (Table 1). Other covariates associated with an increased risk of disease progression were: worse ECOG PS and the presence of liver metastases. On the other hand, higher ERα expression, a longer DFI interval, and having been diagnosed with de novo metastatic disease were associated with better PFS. We found no significant interaction between HER2 status and ERα expression, or type of ET, in affecting PFS/OS (Table 1).

**Fig. 1:**
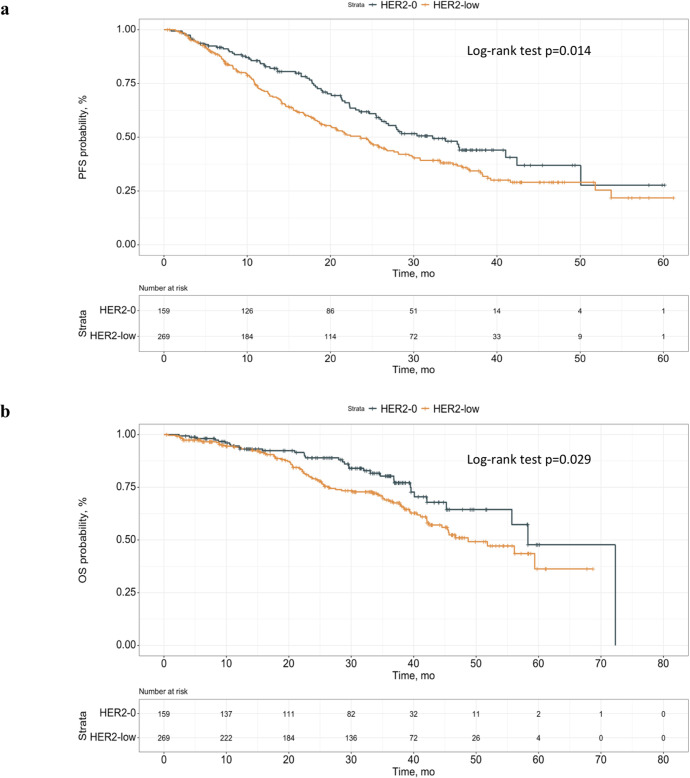
Kaplan–Meier analysis of progression-free survival (a) and overall survival (b) in the HER2-low cohort and HER2-0 cohort. **a** Median Progression-free Survival (PFS) was 23.6 months (95% CI,18.9-28.1) in the HER2-low cohort and 32.3 months (95% CI, 26.1-NA) in the HER2-0 cohort. **b** Median Overall Survival (OS) was 48.7 months (95% CI, 42.5-NA) in the HER2-low cohort and 58.3 months (95% CI, 55.7-NA) in the HER2-0 cohort. HER2 human epidermal growth factor receptor 2, CI confidence intervals, PFS progression free survival.

**Table 1:** Cox proportional hazards multivariable models for progression free survival and overall survival.

	Progression free survival		Overall survival	
	HR (95% CI)	*p*-value*	HR (95% CI)	*p*-value^a^
HER2 status (low vs. 0)^b^	1.42 (1.07–1.89)	0.0163	1.64 (1.08–2.48)	0.0203
Age (continuous)	1.01 (0.82–1.26)	0.9130	1.12 (0.83–1.50)	0.4563
ERα (continuous)^b^	0.91 (0.86–0.96)	0.0010	0.89 (0.82–0.96)	0.0025
ECOG PS, 1 vs. 0	1.41 (1.03–1.92)	0.0060	1.49 (0.98–2.27)	<0.0001
ECOG PS, 2 vs. 0	2.75 (1.34–5.63)		7.09 (3.15–15.95)	
Ki67 (continuous)	1.14 (0.98–1.32)	0.0878	1.08 (0.87–1.33)	0.4988
Number of metastatic sites (continuous)	1.13 (0.97–1.32)	0.1257	1.37 (1.11–1.68)	0.0028
Liver metastases, yes vs. no	1.69 (1.23–2.32)	0.0013	1.15 (0.74–1.79)	0.5316
DFI (continuous)	0.61 (0.47–0.78)	0.0001	0.45 (0.31–0.66)	<0.0001
De novo metastatic, yes vs. no	0.53 (0.35–0.80)	0.0028	0.50 (0.28–0.88)	0.0167
ET, Fulvestrant vs. AIs^2^	1.28 (0.97–1.69)	0.0790	0.98 (0.67–1.43)	0.9177

Abbreviations: *AIs* aromatase inhibitors, *CI* confidence intervals, *DFI* disease-free interval, *HER2* Human Epidermal growth factor Receptor 2, *ECOG PS* Eastern Cooperative Oncology Group Performance Status, *ER* estrogen receptor, *ET* endocrine therapy, *HR* hazard ratio.

^a^p-values were derived by Cox regression models including all the selected variables in the table.

^b^test for interaction: HER2 status and ERα expression (PFS, *p* = 0.0808; OS, *p* = 0.7374), HER2 status and type of ET (PFS, *p* = 0.8904; OS, *p* = 0.6362).

Median patient OS was 56 months in the whole study cohort. Patients with HER2-low disease had significantly worse OS when compared to patients with HER2-0 BC (median OS: 48.7 [42.5-NA] vs 58.3 [55.7–NA] months, *p* = 0.029) (Fig. 1b). Multivariable analysis confirmed an independent association between HER2-low status and worse OS (aHR: 1.64; 95% CI: 1.08–2.48; *p* = 0.0203). (Table 1).

Among 205 patients in whom HER2 status was evaluated both in the primary tumor and in a metastatic lesion, we observed a tumor shift of HER2 status in 72 (35%) patients, including 34 (17%) tumors shifting from HER2-0 to HER2-low status and 34 (17%) tumors shifting from HER2-low to HER2-0 status (Supplementary Table 2, Supplementary Fig. 1). We found an association between HER2-low status and worse PFS and OS in two patient sub-cohorts in which HER2 status was assessed in the primary tumor (Supplementary Fig. 2, Supplementary Table 3) or in a metastatic lesion (Supplementary Fig. 3, Supplementary Table 4).

We showed that HER2-low status is associated with worse PFS and OS in patients with HR + /HER2- aBC treated with first-line ET plus CDK4/6i. Our findings point to HER2 status as a potentially new prognostic biomarker in this clinical context.

Previous studies suggested an association between HER2-low status and worse PFS in HR + /HER2-negative aBC patients receiving ET + CDK4/6i^3–5^. However, these studies were limited by small sample size^4,5^, lack of statistically significant results^5^, or inclusion of patients treated with ET + CDK4/6i as any line of therapy^3,5^. While our findings confirm and expand results of these studies in a larger cohort of patients receiving first-line CDK4/6i, they also provide first evidence that HER2-low status is associated with worse patient OS, which is unlikely to be attributable to the number of subsequent lines of therapy; indeed, in our study patients with HER2-low disease received a higher average number of subsequent lines of systemic therapy when compared to patients with HER2-0 disease after tumor progression to ET + CDK4/6 inhibitors. However, we should be cautious in the interpretation of OS findings in our study; indeed, none of HER2-low patients received T-DXd after progression on ET + CDK4/6 inhibitors. Since T-DXd recently demonstrated to improve the OS of patients with HER2-low aBC^10^, we cannot exclude that the negative prognostic significance of HER2-low status observed in our study may be reversed by the availability of a new and effective systemic treatment. Future studies including patients treated with T-DXd after tumor progression to ET + CDK4/6i will clarify this point.

Previous studies conducted in large series of primary BCs reported on higher ERα/PgR expression in HER2-low as compared to HER2-0 BCs^12^. The fact that we did not observe such association may derive from the lower number of patients included in our study, or by different methods to evaluate ERα/PgR expression across different studies.

The prognostic significance of HER2-low status in BC patients remains a debated topic. Indeed, recently published studies have shown conflicting results. In early-stage BC, HER2-low status was not associated with worse clinical outcomes, and in some of these studies it even revealed a potentially positive prognostic role of HER2-low status^2,13,14^. In patients with aBC, recent reports did not find an association between HER2 status and patient prognosis^6–9^. However, several of these studies were limited by a low number of patients included^7,8^, or by the fact that the prognostic role of HER2 status was evaluated in heterogeneous patient cohorts, i.e., in patients with different tumor biology and receiving different types of systemic therapies^6,9^. A recent, large study from the MD Anderson Cancer Center did not find an association between HER2 status and clinical outcomes in 919 HR + /HER2- aBC patients treated with first-line ET plus CDK4/6 inhibitors^15^. Discrepancies across studies could derive from differences in the population of patients included (e.g., different patient- and tumor-related characteristics), diverse assessment of HER2 status, different clinical management of patients (e.g., different types of backbone ET or CDK4/6i compound used, different subsequent lines of therapy, and in particular T-DXd), or the inclusion of patients with different intrinsic BC subtypes^16^.

Limitations of this study consist in its observational design, the lack of a centralized evaluation of the HER2 status, and the limited follow-up time for survival events.

## Methods

### Study design and enrollment criteria

This was a multicenter, observational, cohort study, approved by the Ethics Committee of Fondazione IRCCS Istituto Nazionale dei Tumori, Milan (INT 138/20). Main enrollment criteria were: diagnosis of HR + /HER2-negative aBC; treatment with first-line ET + CDK4/6i therapy; availability of data about HER2 status. All patients provided written informed consent to take part in the study.

### Study objectives

The primary objective of the study was to investigate the association between HER2 status (low vs. 0) and patient PFS, as defined as the time between initiation of first-line ET + CDK4/6i therapy and disease progression, or patient death from any cause. A secondary objective was to evaluate the association between HER2 status and patient OS, as defined as the time between treatment initiation and patient death from any cause.

### Definition of HER2 status

HER2 status was evaluated in the most recently collected tumor specimen, which could consist in (1) a core biopsy of a metastatic lesion obtained before ET + CDK/4/6i therapy initiation; (2) a core biopsy or surgical primary tumor specimen, if a biopsy of a metastatic lesion was not available.

HER2-low disease was defined by the presence of an IHC score for HER2 of 1 + , or 2+ with negative ISH^1^. HER2-0 status was defined by the presence of an IHC score for HER2 of 0. Estrogen receptor (ERα), Progesteron Receptor (PgR) and Ki-67 expression were evaluated in the same tumor specimen in which HER2 status was assessed.

For p185 IHC, we used Polyclonal Rabbit Anti-Human c-erbB-2 Oncoprotein ref. A0485. Since March 2020, HER2 ISH status was determined with HER2 probe Ventana HER2 DUAL ISH DNA Probe Cod. 800-604. Before March 2020, HER2 ISH probe was Inform HER2 Dual ISH DNA Probe Cocktail.

### Statistical analyses

The association between HER2 status and clinically relevant variables was evaluated through the chi-squared test (for categorical variables) or the Wilcoxon rank sum test (for continuous variables). PFS and OS in patients with HER2-low vs. HER2-0 disease were compared through the log-rank test, and survival curves were represented through the Kaplan–Meier method. Multivariable analyses were performed through Cox regression models, which included clinically relevant covariates along with HER2 status. Based on previous data regarding the correlation between HER2-low status and ERα expression^17^, we also evaluated the interaction between these two variables in affecting PFS. Missing data were imputed through single imputation.

### Reporting summary

Further information on research design is available in the Nature Research Reporting Summary linked to this article.

## Supplementary information

Supplemental material

Reporting Summary

## Data Availability

The data that support the findings of this study are available upon request to the corresponding author, CV.
