# Supplementary material for: Prognostic significance of HER2-low status in HR-positive/HER2-negative advanced breast cancer treated with CDK4/6 inhibitors
Source: NPJ Breast Cancer. 2023 Apr 17;9:27. doi: 10.1038/s41523-023-00534-1 (PMC10110597; doi:10.1038/s41523-023-00534-1)
Supplement: Supplementary file 1 — Supplemental material [file 41523_2023_534_MOESM1_ESM.docx]

**Supplementary Table 1: Baseline patient and tumor characteristics in the whole study cohort and by HER2-status (low vs 0).**

|  | Overall  (n=428) | HER2-0  (n=159) | HER2-low  (n=269) | p-value^1^ |
| --- | --- | --- | --- | --- |
| Age (years)  Median  Q1-Q3  Missing | 63.0  53.8-71.0  - | 63.0  51.5-71.0  - | 63.0  54.0-71.0  - | 0.851 |
| DFI (years)  Median  Q1-Q3  Missing | 5.0  0.75-10.0  8 | 5.0  0-11.0  5 | 5.0  1.0-10.0  3 | 0.897 |
| ERα (%)  Median  Q1-Q3  Missing | 95  90-95  15 | 95  90-95  4 | 95  90-95  11 | 0.964 |
| PgR (%)  Median  Q1-Q3  Missing | 47  3-90  14 | 62  2-90  5 | 40  5-83  9 | 0.116 |
| Ki67 (%)  Median  Q1-Q3  Missing | 21  15-32  22 | 21  14-30  8 | 22  15-35  14 | 0.277 |
| ECOG PS  0  1  2  Missing | 303 (70.8)  107 (25.0)  18 (4.2)  0 | 107 (67.3)  43 (27.0)  9 (5.7)  0 | 196 (72.9)  64 (23.8)  9 (3.3)  0 | 0.344 |
| Menopausal state  Pre-menopausal  Post-menopausal  Missing | 73 (17.1)  355 (82.9)  0 | 30 (18.9)  129 (81.1)  0 | 43 (16.0)  226 (84.0)  0 | 0.527 |
| N. metastatic sites  1-2  >2  Missing | 331 (77.3)  97 (22.7)  0 | 121 (76.1)  38 (23.9)  0 | 210 (78.1)  59 (21.9)  0 | 0.726 |
| Liver metastases  No  Yes  Missing | 333 (77.8)  95 (22.2)  0 | 126 (79.2)  33 (20.8)  0 | 207 (77.0)  62 (23.0)  0 | 0.666 |
| Type of CKD4/6i  Palbociclib  Ribociclib  Abemaciclib  Missing | 291 (68.0)  91 (21.3)  46 (10.7)  0 | 105 (66.1)  36 (22.6)  18 (11.3)  0 | 186 (69.2)  55 (20.4)  28 (10.4)  0 | 0.794 |
| Type of ET  AI  Fulvestrant  Missing | 255 (59.6)  173 (40.4)  0 | 94 (59.1)  65 (40.9)  0 | 161 (59.9)  108 (40.1)  0 | 0.962 |
| Subsequent lines of treatment  Mean  SD  Missing | 1.39  1.68  19 | 1.24  1.49  5 | 1.47  1.77  14 | <0.001 |
| Abbreviations: AI: aromatase inhibitor; CDK4/6inh: Cyclin-Dependent Kinase 4/6 inhibitor; DFI: Disease Free Interval; ECOG PS: Eastern Cooperative Oncology Group Performance Status; HER2, human epidermal growth factor 2; ET: Endocrine Therapy; N: number; Q1-Q3: interquartile range; SD: standard deviation. ^1^ Chi-squared test for categorical variables, Wilcoxon rank sum test for continuous variables | | | | |

**Supplementary Table 2: HER2 status evolution from primary tumor to disease recurrence for patients with both determinations available.**

| Patients with both determinations available | | HER2 status of disease recurrence (%) | | Total |
| --- | --- | --- | --- | --- |
|  |  | 0 | Low |  |
| HER2 status of primary tumor (%) | 0 | 41 (20.1%) | 34 (16.7%) | 75 (36.6%) |
|  | Low | 34 (16.6%) | 92 (44.9%) | 126 (61.5%) |
|  | Positive | 1 (<1%) | 3 (1.5%) | 4 (1.9%) |
| Total | | 76 (37.1%) | 129 (62.9%) | 205 |

205/428 (47.9%) patients had both primary and recurrence tumor HER2 status available, 172 (40.2%) had only primary HER2 status available, 51 (24.9%) had only recurrence tumor HER2 status available, 256 (59.8%) had recurrence tumor HER2 status available.

**Supplementary Table 3: Cox Proportional Hazards Multivariable Models for Progression Free Survival and Overall Survival considering HER2 status of the primary tumor.**

|  | Progression Free Survival | | Overall Survival | |
| --- | --- | --- | --- | --- |
|  | HR (95% CI) | p-value^1^ | HR (95% CI) | p-value^1^ |
| HER2 status (low vs. 0) | 1.30 (0.95-1.76) | 0.0974 | 1.69 (1.08-2.63) | 0.0216 |
| Age (continuous) | 1.04 (0.83-1.31) | 0.7332 | 1.15 (0.84-1.57) | 0.3869 |
| ERα (continuous) | 0.91 (0.86-0.97) | 0.0030 | 0.87 (0.80-0.94) | 0.0004 |
| ECOG PS, 1 vs. 0 | 1.38 (0.98-1.92) | 0.0048 | 1.42 (0.90-2.23) | <0.0001 |
| ECOG PS, 2 vs. 0 | 3.73 (1.57-8.87) |  | 12.28 (4.80-31.41) |  |
| Ki67 (continuous) | 1.17 (0.99-1.37) | 0.0538 | 1.03 (0.82-1.29) | 0.7838 |
| Number of metastatic sites (continuous) | 1.16 (0.98-1.38) | 0.0831 | 1.39 (1.12-1.74) | 0.0031 |
| Liver metastases, yes vs. no | 1.67 (1.19-2.35) | 0.0033 | 1.26 (0.79-1.99) | 0.3366 |
| DFI (continuous) | 0.55 (0.39-0.77) | 0.0006 | 0.41 (0.25-0.67) | 0.0003 |
| De novo metastatic, yes vs. no | 0.43 (0.27-0.69) | 0.0005 | 0.45 (0.24-0.84) | 0.0115 |
| ET, Fulvestrant vs. AIs | 1.23 (0.91-1.66) | 0.1770 | 1.02 (0.68-1.52) | 0.9388 |
| Abbreviations: AIs, aromatase inhibitors; CI, confidence intervals; DFI, disease-free interval; HER2, Human Epidermal growth factor Receptor 2; ECOG PS, Eastern Cooperative Oncology Group Performance Status; ER, estrogen receptor; ET, endocrine therapy; HR, hazard ratio  ^1^ p-values were derived by Cox regression models including all the selected variables in the table | | | | |

**Supplementary Table 4: Cox Proportional Hazards Multivariable Models for Progression Free Survival and Overall Survival considering HER2 status of the metastatic site.**

|  | Progression Free Survival | | Overall Survival | |
| --- | --- | --- | --- | --- |
|  | HR (95% CI) | p-value^1^ | HR (95% CI) | p-value^1^ |
| HER2 status (low vs. 0) | 1.52 (1.01-2.28) | 0.0452 | 2.07 (1.10-3.90) | 0.0248 |
| Age (continuous) | 1.16 (0.86-1.56) | 0.3269 | 1.19 (0.78-1.82) | 0.4251 |
| ERα (continuous) | 0.88 (0.82-0.94) | <0.0001 | 0.88 (0.81-0.97) | 0.0076 |
| ECOG PS, 1 vs. 0 | 1.30 (0.82-2.04) | 0.3787 | 1.40 (0.74-2.66) | 0.0310 |
| ECOG PS, 2 vs. 0 | 1.67 (0.64-4.37) |  | 4.47 (1.45-13.77) |  |
| Ki67 (continuous) | 1.19 (0.98-1.44) | 0.0735 | 1.12 (0.85-1.46) | 0.4298 |
| Number of metastatic sites (continuous) | 1.00 (0.81-1.25) | 0.9433 | 1.48 (1.09-2.01) | 0.0127 |
| Liver metastases, yes vs. no | 1.77 (1.16-2.69) | 0.0079 | 1.01 (0.55-1.84) | 0.9853 |
| DFI (continuous) | 0.72 (0.54-0.96) | 0.0260 | 0.53 (0.33-0.84) | 0.0068 |
| De novo metastatic, yes vs. no | 0.93 (0.47-1.81) | 0.8198 | 0.69 (0.26-1.84) | 0.4561 |
| ET, Fulvestrant vs. AIs | 1.28 (0.88-1.86) | 0.1969 | 0.89 (0.53-1.50) | 0.6661 |
| Abbreviations: AIs, aromatase inhibitors; CI, confidence intervals; DFI, disease-free interval; HER2, Human Epidermal growth factor Receptor 2; ECOG PS, Eastern Cooperative Oncology Group Performance Status; ER, estrogen receptor; ET, endocrine therapy; HR, hazard ratio  ^1^ p-values were derived by Cox regression models including all the selected variables in the table | | | | |

**Supplementary Figure 1: Alluvial plot showing HER2 status evolution from primary tumor to disease recurrence.** The plot illustrates the absolute number of tumor samples in which we observed stable HER2 status in the primary and metastatic tumor specimens, as well as the number of tumor samples in which we observed a shift in HER2 status between the primary and metastatic tumor samples (from HER2-low/HER2-positive to HER2-0, or from HER2-0 to HER2-low).

***
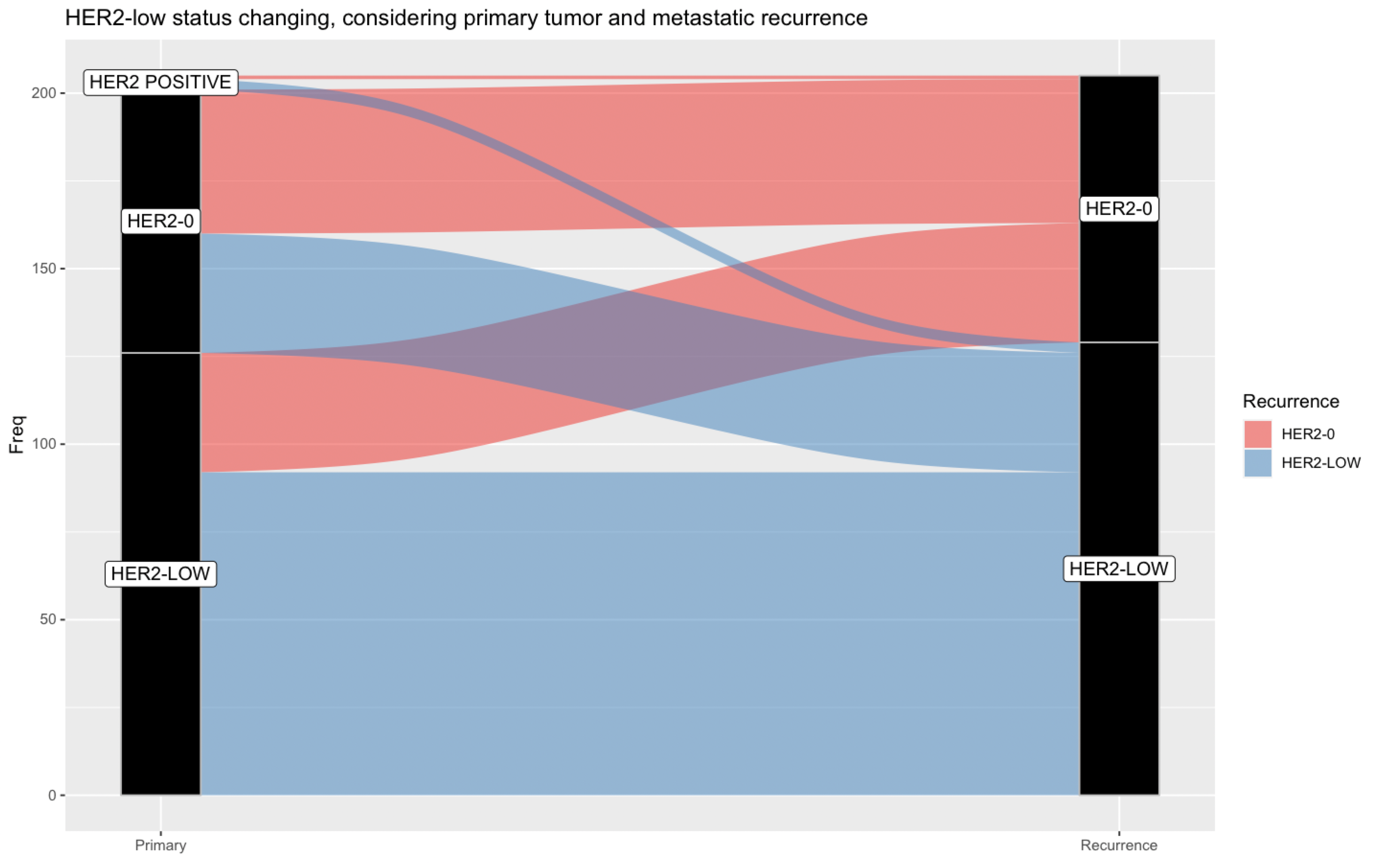
***

**Supplementary Figure 2: Kaplan–Meier Analysis of Progression-free Survival (a) and Overall Survival (b) according to HER2 status in a primary tumor specimen.** (a) Median Progression-free Survival (PFS) was 23.6 months (95% CI,18.9-29.8) in the HER2-low cohort and 28.1 months (95% CI, 24.7-41.0) in the HER2-0 cohort. (b) Median Overall Survival (OS) was 45.7 months (95% CI, 42.5-NA) in the HER2-low cohort and 55.7 months (95% CI, 46.7-NA) in the HER2-0 cohort.

**Supplementary Figure 3: Kaplan–Meier Analysis of Progression-free Survival (a) and Overall Survival (b) according to HER2 status in a metastatic tumor lesion.** (a) Median Progression-free Survival (PFS) was 24.5 months (95% CI,18.6-30.8) in the HER2-low cohort and 35.2 months (95% CI, 27.9-NA) in the HER2-0 cohort. (b) Median Overall Survival (OS) was 48.7 months (95% CI, 42.5-NA) in the HER2-low cohort and 72.3 months (95% CI, 58.3-NA) in the HER2-0 cohort.
